# Systems approaches for synthetic biology: a pathway toward mammalian design

**DOI:** 10.3389/fphys.2013.00285

**Published:** 2013-10-09

**Authors:** Rahul Rekhi, Amina A. Qutub

**Affiliations:** Department of Bioengineering, Rice UniversityHouston, TX, USA

**Keywords:** systems biology, synthetic biology, mammalian cell, computational biology, regenerative medicine, gene circuits, signaling network, multiscale modeling

## Abstract

We review methods of understanding cellular interactions through computation in order to guide the synthetic design of mammalian cells for translational applications, such as regenerative medicine and cancer therapies. In doing so, we argue that the challenges of engineering mammalian cells provide a prime opportunity to leverage advances in computational systems biology. We support this claim systematically, by addressing each of the principal challenges to existing synthetic bioengineering approaches—stochasticity, complexity, and scale—with specific methods and paradigms in systems biology. Moreover, we characterize a key set of diverse computational techniques, including agent-based modeling, Bayesian network analysis, graph theory, and Gillespie simulations, with specific utility toward synthetic biology. Lastly, we examine the mammalian applications of synthetic biology for medicine and health, and how computational systems biology can aid in the continued development of these applications.

## Introduction and overview

Over the past three decades, rapid advances in computational power, subcellular data resolution, and the sophistication of bioengineering design has led to cellular machinery being increasingly controlled for practical application (Buetow, 2005; Cheng, 2007; Vendruscolo and Dobson, 2011). The advent of this field of “synthetic biology” has been touted as a reservoir of novel solutions for many of society's most pressing problems, including challenges in computing, health, and regenerative medicine (Gersbach et al., 2007; Lu et al., 2009; Ruder et al., 2011). For instance, the creation of the first-ever genetic toggle switch and the repressilator by synthetic biologists at the turn of the century allowed for an unprecedented degree of cellular control—and, in the case of the former, a digital state that could lay the groundwork for organic computing (Elowitz and Leibler, 2000; Gardner et al., 2000). In subsequent years, biologists constructed oscillators (capable of biological timekeeping), pulse generators (for transcellular signal transmission), and even signaling filters (for cellular signal processing) through carefully mapped gene circuits (Basu et al., 2004, 2005; Stricker et al., 2008; Khalil and Collins, 2010).

However, while each of these individual discoveries led to numerous applications of genetic engineering in biomedicine, we still lack tools with the robustness required for transformative applications. For instance, true “plug-and-play” cellular machines remain a work in progress, in part due to the heterogeneity and adaptability of biological networks (Kobayashi et al., 2004; Arkin, 2008). The routine engineering of mammalian cells, too, is still a distant possibility (Khalil and Collins, 2010). Because synthetic biology has largely been applied to microbes due to mammalian cell complexity, its impact on medicine has been limited.

Achieving these benchmarks is admittedly easier said than done. Whereas the promises and potential of the synthetic biology field lie in characterizing the cellular alphabet, the puzzle of words and sentences that define cell signaling and behavior currently present a higher order of complexity (Endy, 2005). Moreover, the field of synthetic biology is still in its infancy, compared to the equivalent of “the Wright brothers … putting pieces of wood and paper together” (Kwok, 2010). Some leading researchers have even suggested that “the complexity of synthetic biological systems over the past decade has reached a plateau” (Purnick and Weiss, 2009).

One way biologists have started to reinvigorate the field is through advances in combinatorial logic-based circuits (Lu et al., 2009; Wang et al., 2011; Michelotti et al., 2012; Wang and Buck, 2012). These formalisms possess the distinct advantages of providing a standardized framework that is adaptable across levels of abstraction as well as dynamical properties that can be estimated and combined by straightforward mathematical operations. Showing early progress, combinatorial logic-based circuits have been designed into sophisticated information processing tools in clonal mammalian cells like HeLa and MCF-7 (Xie et al., 2011; Nevozhay et al., 2013). However, noise, heterogeneity, complexity of structure, and time-dependent rewiring across biological scales limit the degree of control enabled by these experimental methods (Purnick and Weiss, 2009; Kwok, 2010). We propose that these challenges can be tackled by capitalizing on advances in computational systems biology that are uniquely valuable for synthetic cell design. We argue that a new perspective on the role of systems modeling in synthetic biology can promote the development of new therapies for human health by enabling the complex design capability required for mammalian cell engineering.

## Computational techniques and advances: systems biology applications

**Figure 1:**
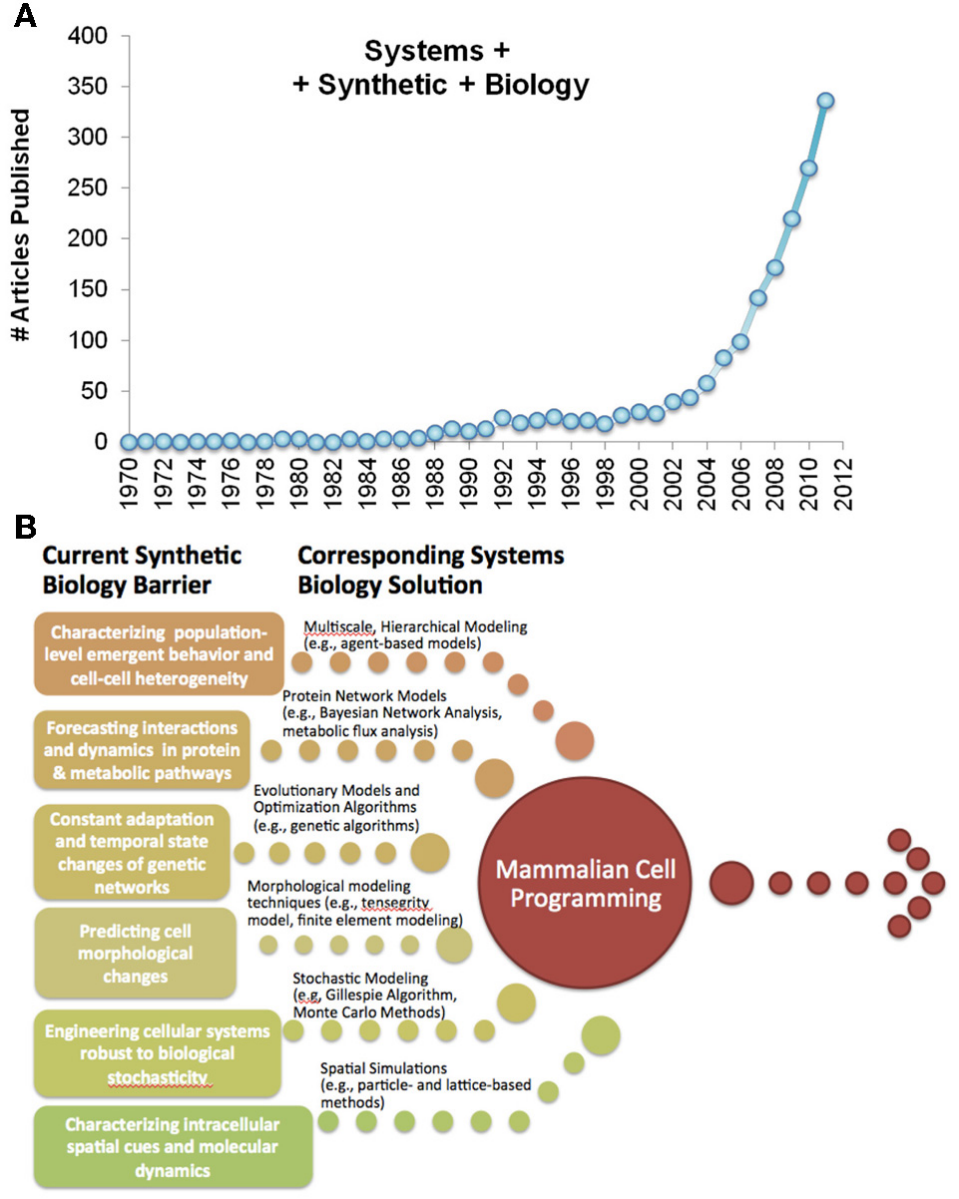
**(A)** PUBMED references to Systems and Synthetic biology over the last four decades. **(B)** Recent advances in systems biology can be applied toward surmounting specific limitations to existing synthetic biology, paving the way to mammalian cell engineering.

Computational methods are widely employed within synthetic biology as design tools, providing simulations of bioengineered systems in advance of their cellular assembly (Chandran et al., 2009; Ellis et al., 2009; Purnick and Weiss, 2009; Smolke and Silver, 2011) (Figure 1). Historically, these coupled computational-experimental approaches have contributed to many of the “milestone” discoveries in the field over the past two decades (Table 1). However, modeling used in synthetic biology until now has been generally limited to biocircuits and control systems, in part because the field emerged from genetic engineering where circuit representations are common (Mukherji and van Oudenaarden, 2009). The consequence of this limited paradigm is that significant advances in human health from this field remain out of reach, as gene circuit models prove to be increasingly insufficient for characterizing mammalian cell behavior (Purnick and Weiss, 2009).

**Table 1:** **Synthetic biology milestones employing computational methods, as well as those that were built conceptually from computational paradigms**.

**Synthetic biology milestone**	**Computational method employed**	**Year**	**References**
Bacterial toggle switch	Receptor-ligand binding kinetics, gene circuit analysis, analog computing	2000	Kramer et al., 2004
Repressilator	Receptor-ligand binding kinetics, stochastic simulation, gene circuit analysis	2000	Elowitz and Leibler, 2000
Stochastic gene expression	Stochastic noise modeling	2002	Elowitz et al., 2002
Programmed bacterial population control	Logistic ODE kinetics, gene circuit analysis	2004	You et al., 2004
Mammalian transgene switch	Gene circuit analysis	2004	Kramer et al., 2004
Programmed pattern formation	Logistic ODE kinetics, statistical analysis, gene circuit analysis	2005	Basu et al., 2005
Engineered yeast produce artemesinin	Gene circuit analysis	2006	Ro et al., 2006
Engineered bacteria target cancer by expressing invasion	Gene circuit analysis	2006	Anderson et al., 2006
RNAi logic circuits	Boolean evaluation, gene circuit analysis	2007	Xie et al., 2011
Creation of logic gates	Gene circuit analysis, boolean operator models	2008	Win and Smolke, 2008
Bacterial edge detection	Electronic signal processing, analog computing, gene circuit analysis	2009	Tabor et al., 2009
Implementation of artificial genome	Gene circuit analysis	2010	Gibson et al., 2010
Whole-cell computational model	Flux balance analysis, poisson processes, ODEs, receptor-ligand kinetics, stochastic simulation, boolean operators	2012	Karr et al., 2012

In the aggregate, this “insufficiency” stems from a set of core properties of biological systems that current synthetic approaches do not fully capture: (1) *scale*, with the need to elicit controlled behavior across cell, tissue, and organ levels (Miller et al., 2012); (2) *simultaneity*, as defined by the highly networked nature of cell signaling (Jeong et al., 2000, 2001; Marcotte, 2001); (3) *state adaptation dynamics*, or the non-linear temporal fluctuations of such networks (Slusarczyk et al., 2012); (4) *shape*, due to the relevance of cell morphology in defining environmental interactions (Ben-Ze'ev et al., 1988; Singhvi et al., 1994); (5) *stochasticity*, with noise and randomness being significant determinants of cellular behavior (Thattai and van Oudenaarden, 2001; Pedraza and van Oudenaarden, 2005; Chopra and Kamma, 2006; Purnick and Weiss, 2009); and (6) *spatial dependencies*, both intracellular and extracellular in nature (Andrianantoandro et al., 2006). With the present focus on microbial engineering, many of these characteristics can be safely neglected; at mammalian levels of complexity, they render the behavior of synthetic systems difficult to predict *a priori*.

Although these challenges are manifold, they are not insurmountable. The answers may lie in systems biology. This computational discipline seeks to shift the basic molecular biology paradigm from isolation to coordination: from characterizing individual components of cell behavior to analyzing how these components function in tandem (Kitano, 2002a,b). Accordingly, systems bioengineers bring a diverse array of computational modeling techniques—drawing on mathematics, computer science, and engineering—to bear on questions of both mechanism and design at the cell and tissue levels (Kitano, 2001; Alon, 2007). In doing so, the field provides computational tools to characterize behavioral patterns at the cellular level that will be the building blocks of more sophisticated synthetic design. Systems biology approaches are particularly powerful in characterizing cell–cell interactions across scales, such as in capillary patterning and organ development, where the gene circuits approach in synthetic biology has proven limited in capturing adaptation, cellular heterogeneity and spatial hierarchy (Yingling et al., 2005; Qutub et al., 2009; Long et al., 2013). As such, many of the challenges to applying synthetic biology toward controlling mammalian tissue can be addressed in part by methods and techniques that are well-developed in systems bioengineering. Here, we discuss each of these roadblocks categorically, with the associated tools to address them.

### Scale: hierarchical, agent-based modeling, and rule-based formalisms

Characterizing population-level emergent behavior and cell–cell heterogeneity has long been recognized as a principal goal and challenge in synthetic biology (Canton et al., 2008; Neumann and Neumann-Staubitz, 2010; Young and Alper, 2010). Traditional synthetic designs have assumed identical expression patterns across a cell population, as the standard biocircuit framework does not permit the simulation of cell behavioral variability (Elowitz et al., 2002; Ozbudak et al., 2002; Blake et al., 2003; You et al., 2004). Moreover, the limited capacity for gene circuit models to characterize emergent behavior—defined formally as patterns that emerge from a myriad of relatively simple interactions—inhibits scale-dependent design (Benner and Sismour, 2005). As a mammalian example, if intricate cerebral function results from the coordinated function of millions of individual neurons, any synthetic design applied to the brain must first require accurate simulations of how neural cell-level changes manifest on the cerebral tissue-level. Deriving such scale-driven causal links from observed principles is non-trivial at best.

One systems biology method to address the research challenge of emergence in biology is agent-based modeling. The approach has a simple premise: such systems exhibit emergent behavior that arises from the interactions between individual actors (or agents) and, consequently, would be impossible to know *a priori* (Chandran et al., 2009). An agent is defined here as a discrete entity that has behavior, can adapt, carries “genetic codes,” holds variables and data, is governed by individual rules, and is spatially defined. Fundamentally, this class of modeling method diverges from biocircuit models, which typically characterize fluctuations in state variables governed by differential relationships. Supplanting the latter's top-down, intracellular perspective with the former's bottom-up, multi-scale viewpoint permits the simulation of heterogeneity while eliminating the need to derive inter-scale relationships beforehand (Chandran et al., 2009).

Notably, agent-based modeling encompasses a broad range of variations in implementation, rather than any specific algorithm or rule-set. Existing libraries, such as MASON, Repast, and Swarm, allow for the construction of multi-scale agent-based models atop adaptable frameworks, facilitating their use by synthetic biologists with limited prior exposure to the technique. This methodology has been employed toward modeling brain capillary regeneration (Long et al., 2013), immunological and inflammatory responses (Bailey et al., 2007; Chandran et al., 2009; Pothen et al., 2013), and cancer progression (Wang et al., 2009; Basanta et al., 2012; Wodarz et al., 2012; Walker and Southgate, 2013), among other topics. Within synthetic biology specifically, agent-based models have also simulated tissue development, tissue formation, and microbial chemotaxis (Endler et al., 2009).

Similarly rule-based formalisms are also being applied to coarse-grain patterns in chemical-kinetic models (Feret et al., 2009; Yang et al., 2010), providing scalable tools to describe complex interactions in cellular systems that begin at the molecular level.

### Simultaneity: graph theory and network analysis

Forecasting interactions and dynamics in protein and metabolic pathways is crucial for fine-tuned control of mammalian synthetic bioengineering. Whereas traditional kinetic- and gene circuit-based methods use simplified pathways to represent these signaling dynamics, in many cases the relationships between molecules are highly non-linear (Marcotte, 2001) and multiplex, i.e., multiple inputs combine to a single output. Signals that propagate from A->B->C at regular intervals are rare; more common are those for which such variations as A->C<->B and B->A->C->A dictate the targeted result, with time- and state-dependent transitions (Kestler and Kühl, 2008). Common, too, are linkages between parallel molecular pathways that each simultaneously affect the output of the other (Jeong et al., 2000, 2001). These oscillations render more complex cell pathways intractable for traditional biocircuit methods, which are generally based on small set of ordinary differential equations (ODEs) (Kestler and Kühl, 2008).

Several types of network models allow for better predictive simulation of these multiplex interactions. Graph methods, for example, are a class of models that represent pathway components as networked nodes, and graph-based approaches have been used to model cellular machinery including genes, proteins and other subcellular compartments (Ma'ayan et al., 2005; Pe'er, 2005). The interactions between components are drawn as edge connections between the relevant nodes (Ma'ayan et al., 2005). Graph-based models vary in implementation to capture different kinds of molecular relationships (e.g., Boolean gene expression, stochastic transitions between molecular states), but are all particularly adept at identifying complex network modules, or certain structural features that “dominate” the behavior of the larger network. In mammalian cells, as an example, researchers have had early success in characterizing the dynamics of key feedforward modules and motifs, helping to enable the circuit design of adaptive gene expression (Bleris et al., 2011).

One common type of acyclic graph method, known as Bayesian Network Analysis, is a form of directed statistical modeling designed to capture conditional dependencies between probabilistic events (Pe'er, 2005). In a Bayesian network model, probabilities define the relationship between the current node and its predecessor or parent in a graph (Alterovitz et al., 2007). Markov models are another network-based technique that can provide a framework to describe molecular or cellular states and the weighted probability of transitioning between them. The power of these methods lies in their ability to facilitate the reverse engineering of multiplex networks based on molecular expression, molecular activity and/or cell behavior data, serving as a precursor to synthetic modifications of existing molecular pathways (Barnes et al., 2011). However, for gene or protein pathways with more complex topology—such as those examples offered above—cyclic graph models might be necessary, for which a variety of analytical tools and approaches are described by computational biologists in the literature (particularly from research on neural networks) (Bianchini et al., 2006; Scarselli et al., 2009; Bowsher, 2010; Bonnet et al., 2013).

### State adaptation dynamics: evolutionary models, optimization algorithms

In parallel with the above techniques, another suite of computational methods permits not only the analysis of cellular pathways, but also directly facilitates their synthetic design. Known as evolutionary algorithms, these methods can predict state changes in the behavior of signaling pathways over time, through adaptation or random mutation, by modeling this rewiring directly (Hallinan et al., 2010; Chen et al., 2011; Mobashir et al., 2012). In the same vein, these methods allow for the *de novo* construction and optimization of genetic networks by way of simulation (Bloom and Arnold, 2009), “evolving” a set of viable pathway designs that meet the specified constraints (Hallinan et al., 2010). Though these algorithms vary in construction, a subset of methods known as genetic algorithms—in which populations of potential networks “compete” against each other—are of particular utility to synthetic biologists due to their ease-of-implementation (Mitchell, 1998). Many alternative optimization techniques exist, e.g., simulated annealing, hill climbing, and gradient descent, which can be applied to optimize synthetic network architectures and the design of synthetic constructs (Zomaya and Kazman, 2010). In addition to these, combinatorial “tuning” strategies have been successfully applied toward model-guided, programmable control of gene expression in mammalian cells via RNAi (Beisel et al., 2008). A unique advantage of evolutionary and optimization algorithms is their ability to (A) be applied broadly to many forms of models, including ODEs and rule-based simulations and (B) generate a diverse array of functional network topologies.

### Shape: morphological modeling and computational cell phenotyping

Thus, far, synthetic biology research has largely omitted studies on cell shape. The few exceptions in the literature focus on morphological properties as reporters for specific signaling cascades or to control specific spatial features (Yeh et al., 2007; Tanaka and Yi, 2009). For instance, one recent work described controlled shape changes of synthetic yeast cells (Tanaka and Yi, 2009). Rather than modeling how a gene circuit would induce specific cell morphology *a priori*, the study's authors varied α-factor pathway inputs to observe shape changes until the desired shape was achieved—in this case, one that upregulated the formation of mating projections (Tanaka and Yi, 2009). Another study scored filopodial and lamellipodial phenotypes as indicators for successful synthetic rewiring of Rho GTPase signaling (Yeh et al., 2007).

Despite the few studies in this area, cell morphology is often a characteristic of central importance to synthetic biology experiments. For instance, synthetic systems seeking to modulate cell–cell interactions must necessarily account for morphological and spatial-dependent interactions between cells (Ben-Ze'ev et al., 1988; Singhvi et al., 1994). These membrane adjacency and receptor localization are drivers of pathways like Delta-Notch signaling, in which a signaling cascade is triggered by the binding of two transmembrane proteins on adjacent cells (Appel et al., 2001). Moreover, cell behavior—and at a higher scale, tissue functionality—is often predicated on geometry (Haeuptle et al., 1983). For example, optimizing a synthetic cell for metabolic filtration necessitates that its membrane surface area be maximized for nutrient exchange, such as through inward folds (Gahan, 2005). Doing so requires leveraging computational modeling to predict three-dimensional shape response to changes in genetic circuit design.

Examples of methods for geometrical-rendered modeling of cells include tensegrity models, Voronoi-based simulations, and molecular dynamics models. The concept of “tensegrity” stems from geodesic design, in which an object's shape is maintained through the joint effect of structural members in continuous tension and those in discontinuous compression (Huang et al., 2006). Though abstract in concept, computational models of tensegrity have been demonstrated to approximate cell shape and mechanics, providing a representation for simulating cell morphology *in vitro* (Huang et al., 2006). Tensegrity principles have been used to represent cytoskeletal elements, allowing for changes in these proteins induced by regulatory networks (e.g., focal adhesion kinases) to be assessed for their effects on cell shape (Kardas et al., 2013). An alternative geometrical model is the Voronoi diagram, a mathematical concept of dividing space into distinct regions based on proximity to initial seed points. Voronoi diagrams provide a useful means of constraining complex cell shapes into adjacent spatial tessellations, a technique particularly useful to study patterning at the cell population- or tissue-level (Schaller and Meyer-Hermann, 2005; Luengo-Oroz et al., 2008). Lastly, molecular dynamics simulations of cell shape represent cells as collections of individual molecules in Newtonian motion, either abstractly (as particles) or concretely (as cytoskeletal elements), to model an agglomerated cellular structure at high resolution—albeit at greater computational cost (Rapaport, 2004; Pfaendtner et al., 2010).

Linking geometric-based models to gene network simulations offers the opportunity to guide synthetic biocircuit design *in silico* such that specific cell morphologies can be engineered. Previously, this method has led to a complete representation of osteocyte cytoskeleton dynamics (Kardas et al., 2013). In conjunction, computational cell phenotyping enables changes in morphology to be quantitatively measured and tracked, such that the desired design can be achieved. Phenotyping techniques couple high-fidelity cell imaging with processing metrics to parse shape information (Chung et al., 2008; Sozzani and Benfey, 2011; Ryan et al., 2013). These shape metrics can facilitate the computer-aided design of synthetic networks.

### Stochasticity: gillespie algorithm and monte carlo methods

Perhaps the most significant research challenge in synthetic bioengineering is enabling the design of cellular systems that are robust to biological stochasticity (Chopra and Kamma, 2006; Purnick and Weiss, 2009). Existing gene circuit models are largely deterministic, behaving in highly reproducible ways. These models, as alluded to previously, present regulatory networks as homogeneous concentrations of molecules modulated by parameterized rate constants through coupled differential equations.

Yet there exists increasing evidence that biological networks and intracellular behavior are innately stochastic (Thattai and van Oudenaarden, 2001). Whereas noise effects are often assumed to be negligible at the population level, noise can play a significant role at the single-cell level, e.g., where a small number of molecular interactions may trigger a cascade of downstream protein signaling (Thattai and van Oudenaarden, 2001; Pedraza and van Oudenaarden, 2005). Furthermore, research indicates the phenomenon of noise propagation, in which cell-level stochasticity can accrue at the population-level to create emergent behavior that deviates substantially from the desired target, a phenomena recently documented in *E. Coli*, leading to a loss of synchrony between cells (Hooshangi et al., 2005; Hornung and Barkai, 2008). Such studies suggest that complex synthetic systems cannot be engineered without first accounting for stochasticity in the circuit design.

Fortunately, there exist a wide variety of computational techniques to capture and predict this biological stochasticity at the systems level. One specific approach, known as the Gillespie Algorithm, rejects the deterministic ODE approach of modeling chemical-kinetics in favor of stochastic representations of molecular interactions (Gillespie, 2007). This algorithm explicitly simulates each “reaction” (or interaction event) along a network, with the probability of a successful “reaction” dependent on both the rate properties and a random walk (Gillespie, 2007). For synthetic biology applications, these reactions can be defined as discrete regulatory steps along a specific gene circuit, allowing the effects of noise along the circuit to be well-characterized.

The Gillespie algorithm belongs to a larger class of stochastic modeling techniques known as Monte Carlo methods, which can be adapted to suit the needs of a specific biocircuit design (Athale, 2001). Monte Carlo methods, while varied in implementation, share the property of employing random simulations over many iterations to quantify properties of biological systems.

### Spatial dependencies: particle- and lattice-based methods

Traditional synthetic biology designs are based on assumptions of biochemically homogenous cell interiors, but for gene circuit designs of higher complexity, this set of assumptions is unlikely to hold (Agapakis et al., 2012). Often, the spatial information associated with a protein or pathway inside the cell can influence the end-behavior of a molecular network (Agapakis et al., 2012). In addition to variations in metabolic conditions (e.g., pH levels), spatial cues can also present as receptor- or organelle- localization, intracellular polarity, and even topological sequestration (Harold, 1991; Roze et al., 2011; Lee et al., 2012). Characterizing intracellular spatial dependences and molecular dynamics becomes particularly important in mammalian cells, for which fine spatial organization of regulatory pathways is commonplace.

To this end, particle- and lattice-based computational techniques can be employed to model spatial systems within a synthetic cell (Spicher et al., 2011; Klann and Koeppl, 2012). Rather than simulate bulk flow, particle-based models track molecules separately and in discrete quantities (Takahashi et al., 2005), as alluded to above in the description of molecular dynamic models (see the *Shape* section). In systems biology, such methods have already been applied toward characterizing single-cell gradient sensing in the presence of multiple competitive ligands (Liou and Chen, 2012). Particle models could be similarly applicable to synthetic biology in engineering mammalian cells to function as fine-tuned hypoxic or nitric oxide sensors, in an effort to minimize effects of ischemic stroke—to name just one instance.

The complexity of particle models is mitigated by the availability of open source simulators, including E-Cell and ChemCell (Klann and Koeppl, 2012). Many of these implementations also allow particle simulations to be combined with models of other classes. As an example, a spatial derivative of the Gillespie algorithm can integrate stochastic modeling with space-dependent computation (Takahashi et al., 2005).

Spatial modeling can also be performed using PDE models; examples include gene circuits defining chemical diffusion-mediated interactions between localized cell populations (Song et al., 2009). In other applications to synthetic biology, these spatial techniques have been combined with mechanistic models, such as kinetic RNA folding simulations, to provide fine-tuned control of gene expression along a specific component of a regulatory pathway (Carothers et al., 2011). PDE formalisms offer relative simplicity of construction compared to other spatiotemporal methods, with the caveat of not being well-suited to highly heterogeneous spatial environments.

## Applications for mammalian cells and human health

Until now, the overwhelming focus of research and progress in synthetic biology has been on prokaryotic cells: mostly bacteria (commonly *E. Coli*) and assorted microbes. This is a natural consequence of the knowledge gap described previously; prokaryotic cells are orders of magnitude simpler than eukaryotic ones—not to mention easier to manipulate. They could be said to represent the “crawling” stage of synthetic biology. However, if the ultimate goal of the discipline is to uncover novel therapeutic targets and treatments in biomedicine, such strict characterization of non-mammalian systems will restrain our ability to advance human health. In the end, we must learn to walk.

To do so means confronting the complexity that the *in vivo* mammalian system brings. Methods already employed in systems biology to characterize this complexity can open up the boundaries of modern medicine. As an example, it is not difficult to imagine a future where computational models enable the design of synthetic neural progenitor cells programmed to promote recovery post-ischemic stroke. To foster an era of personalized medicine, this potential could revolutionize the manner in which we approach tissue engineering: cells grown *en masse*, and then programmed to meet the specific needs of the patient. Moreover, such customizable cells would permit targeted regeneration to a degree that simple stem cell treatments cannot achieve. Such innovations, while distant, are attainable, but they necessitate the coupling of systems approaches with synthetic biology.

## Concluding comments

Bringing the sister disciplines of synthetic and systems biology closer together could recast the gene circuit paradigm, and enhance our ability to engineer and program cells for applications across energy, computing and biomedicine. Leveraging a computational toolkit refined by systems biologists for the last half-century offers a unique catalyst that to help pave the future of synthetic biology.

### Conflict of interest statement

The authors declare that the research was conducted in the absence of any commercial or financial relationships that could be construed as a potential conflict of interest.

## References

[B1] Agapakis C. M. Boyle P. M. Silver P. A. (2012). Natural strategies for the spatial optimization of metabolism in synthetic biology. Nat. Chem. Biol. 8, 527–535 10.1038/nchembio.975 22596204

[B2] Alon U. (2007). An Introduction to Systems Biology: Design Principles of Biological Circuits. Chapman and Hall/CRC Press.

[B3] Alterovitz G. Liu J. Afkhami E. Ramoni M. F. (2007). Bayesian methods for proteomics. Proteomics 7, 2843–2855 10.1002/pmic.200700422 17654463

[B4] An G. Mi Q. Dutta−Moscato J. Vodovotz Y. (2009). Agent−based models in translational systems biology. Wiley Interdiscip. Rev. Syst. Biol. Med. 1, 159–171 10.1002/wsbm.45 20835989 PMC3640333

[B5] Anderson J. C. Clarke E. J. Arkin A. P. Voigt C. A. (2006). Environmentally controlled invasion of cancer cells by engineered bacteria. J. Mol. Biol. 355, 619–627 10.1016/j.jmb.2005.10.076 16330045

[B6] Andrianantoandro E. Basu S. Karig D. K. Weiss R. (2006). Synthetic biology: new engineering rules for an emerging discipline. Mol. Syst. Biol. 2, 10–13 10.1038/msb4100073 16738572 PMC1681505

[B7] Appel B. Givan L. A. Eisen J. (2001). Delta-Notch signaling and lateral inhibition in zebrafish spinal cord development. BMC Dev. Biol. 1:13 10.1186/1471-213X-1-13 11495630 PMC37243

[B8] Arkin A. (2008). Setting the standard in synthetic biology. Nat. Biotechnol. 26, 771–773 10.1038/nbt0708-771 18612298

[B9] Athale C. (2001). Monte Carlo cell simulations. Genome Biol. 3:reports2001 10.1186/gb-2001-3-1-reports2001

[B10] Bailey A. M. Thorne B. C. Peirce S. M. (2007). Multi-cell agent-based simulation of the microvasculature to study the dynamics of circulating inflammatory cell trafficking. Ann. Biomed. Eng. 35, 916–936 10.1007/s10439-007-9266-1 17436112

[B11] Barnes C. P. Silk D. Sheng X. Stumpf M. P. H. (2011). Bayesian design of synthetic biological systems. Proc. Natl. Acad. Sci. U.S.A. 108, 8645–8650 10.1073/pnas.1017972108 21876136 PMC3174594

[B12] Basanta D. Gatenby R. A. Anderson A. R. A. (2012). Exploiting evolution to treat drug resistance: combination therapy and the double bind. Mol. Pharm. 9, 914–921 10.1021/mp200458e 22369188 PMC3325107

[B13] Basu S. Gerchman Y. Collins C. H. Arnold F. H. Weiss R. (2005). A synthetic multicellular system for programmed pattern formation. Nature 434, 1130–1134 10.1038/nature03461 15858574

[B14] Basu S. Mehreja R. Thiberge S. Chen M.-T. Weiss R. (2004). Spatiotemporal control of gene expression with pulse-generating networks. Proc. Natl. Acad. Sci. U.S.A. 101, 6355–6360 10.1073/pnas.0307571101 15096621 PMC404049

[B15] Beisel C. L. Bayer T. S. Hoff K. G. Smolke C. D. (2008). Model-guided design of ligand-regulated RNAi for programmable control of gene expression. Mol. Syst. Biol. 4, 1–13 10.1038/msb.2008.62 18956013 PMC2583087

[B16] Benner S. A. Sismour A. M. (2005). Synthetic biology. Nat. Rev. Genet. 6, 533–543 10.1038/nrg1637 15995697 PMC7097405

[B17] Ben-Ze'ev A. Robinson G. S. Bucher N. L. Farmer S. R. (1988). Cell–cell and cell-matrix interactions differentially regulate the expression of hepatic and cytoskeletal genes in primary cultures of rat hepatocytes. Proc. Natl. Acad. Sci. U.S.A. 85, 2161–2165 10.1073/pnas.85.7.2161 3353374 PMC279949

[B18] Bianchini M. Gori M. Sarti L. Scarselli F. (2006). Recursive processing of cyclic graphs. IEEE Trans. Neural Netw. 17, 10–18 10.1109/TNN.2005.860873 16526472

[B19] Blake W. J. KAErn M. Cantor C. R. Collins J. J. (2003). Noise in eukaryotic gene expression. Nature 422, 633–637 10.1038/nature01546 12687005

[B20] Bleris L. Xie Z. Glass D. Adadey A. Sontag E. Benenson Y. (2011). Synthetic incoherent feedforward circuits show adaptation to the amount of their genetic template. Mol. Syst. Biol. 7, 1–12 10.1038/msb.2011.49 21811230 PMC3202791

[B21] Bloom J. D. Arnold F. H. (2009). In the light of directed evolution: pathways of adaptive protein evolution. Proc. Natl. Acad. Sci. U.S.A. 106, 9995–10000 10.1073/pnas.0901522106 19528653 PMC2702793

[B22] Bonnet E. Calzone L. Rovera D. Stoll G. Barillot E. Zinovyev A. (2013). BiNoM 2.0, a Cytoscape plugin for accessing and analyzing pathways using standard systems biology formats. BMC Syst. Biol. 7:18 10.1186/1752-0509-7-18 23453054 PMC3646686

[B23] Bowsher C. G. (2010). Stochastic kinetic models: dynamic independence, modularity and graphs. Ann. Stat. 38, 2242–2281 10.1214/09-AOS779 21278808 PMC3027064

[B24] Buetow K. H. (2005). Cyberinfrastructure: empowering a “third way” in biomedical research. Science 308, 821–824 10.1126/science.1112120 15879210

[B25] Canton B. Labno A. Endy D. (2008). Refinement and standardization of synthetic biological parts and devices. Nat. Biotechnol. 26, 787–793 10.1038/nbt1413 18612302

[B26] Carothers J. M. Goler J. A. Juminaga D. Keasling J. D. (2011). Model-driven engineering of RNA devices to quantitatively program gene expression. Science 334, 1716–1719 10.1126/science.1212209 22194579

[B27] Chandran D. Copeland W. B. Sleight S. C. Sauro H. M. (2009). Mathematical modeling and synthetic biology. Drug Discov. Today Dis. Models 5, 299–309 10.1016/j.ddmod.2009.07.002 PMC5102263 27840651

[B28] Chen B.-S. Hsu C.-Y. Liou J.-J. (2011). Robust design of biological circuits: evolutionary systems biology approach. J. Biomed. Biotechnol. 2011:304236 10.1155/2011/304236 22187523 PMC3237015

[B29] Cheng T. (2007). Moore's law meets the life sciences. IEEE Des. Test Comput. 24, 4 10.1109/MDT.2007.23

[B30] Chopra P. Kamma A. (2006). Engineering life through synthetic biology. In Silico Biol. 6, 401–410 17274769

[B31] Chung K. Crane M. M. Lu H. (2008). Automated on-chip rapid microscopy, phenotyping and sorting of C. elegans. Nat. Methods 5, 637–643 10.1038/nmeth.1227 18568029

[B32] Ellis T. Wang X. Collins J. J. (2009). Diversity-based, model-guided construction of synthetic gene networks with predicted functions. Nat. Biotechnol. 27, 465–471 10.1038/nbt.1536 19377462 PMC2680460

[B33] Elowitz M. B. Leibler S. (2000). A synthetic oscillatory network of transcriptional regulators. Nature 403, 335–338 10.1038/35002125 10659856

[B34] Elowitz M. B. Levine A. J. Siggia E. D. Swain P. S. (2002). Stochastic gene expression in a single cell. Science 297, 1183–1186 10.1126/science.1070919 12183631

[B35] Endler L. Rodriguez N. Juty N. Chelliah V. Laibe C. Li C. (2009). Designing and encoding models for synthetic biology. J. R. Soc. Interface 6, S405–S417 10.1098/rsif.2009.0035.focus 19364720 PMC2843962

[B36] Endy D. (2005). Foundations for engineering biology. Nature 438, 449–453 10.1038/nature04342 16306983

[B37] Feret J. Danos V. Krivine J. Harmer R. Fontana W. (2009). Internal coarse-graining of molecular systems. Proc. Natl. Acad. Sci. U.S.A. 106, 6453–6458 10.1073/pnas.0809908106 19346467 PMC2672529

[B38] Gahan P. B. (2005). Life: the science of biology (7th edn) W. K. Purves, D. Sadava, G. H. Orians and H. C. Heller, W. H. Freeman and Co, 1121 pp., ISBN 0-7167-9856-5 (2004). Cell Biochem. Funct. 23, 221 10.1002/cbf.1179

[B39] Gardner T. S. Cantor C. R. Collins J. J. (2000). Construction of a genetic toggle switch in *Escherichia coli*. Nature 403, 339–342 10.1038/35002131 10659857

[B40] Gersbach C. A. Phillips J. E. García A. J. (2007). Genetic engineering for skeletal regenerative medicine. Annu. Rev. Biomed. Eng. 9, 87–119 10.1146/annurev.bioeng.9.060906.151949 17425467

[B41] Gibson D. G. Glass J. I. Lartigue C. Noskov V. N. Chuang R.-Y. Algire M. A. (2010). Creation of a bacterial cell controlled by a chemically synthesized genome. Science 329, 52–56 10.1126/science.1190719 20488990

[B42] Gillespie D. T. (2007). Stochastic simulation of chemical kinetics. Annu. Rev. Phys. Chem. 58, 35–55 10.1146/annurev.physchem.58.032806.104637 17037977

[B43] Haeuptle M. T. Suard Y. L. Bogenmann E. Reggio H. Racine L. Kraehenbuhl J. P. (1983). Effect of cell shape change on the function and differentiation of rabbit mammary cells in culture. J. Cell Biol. 96, 1425–1434 10.1083/jcb.96.5.1425 6841452 PMC2112657

[B44] Hallinan J. S. Misirli G. Wipat A. (2010). Evolutionary computation for the design of a stochastic switch for synthetic genetic circuits. Conf. Proc. IEEE Eng. Med. Biol. Soc. 2010, 768–774 10.1109/IEMBS.2010.5626353 21095906

[B45] Harold F. M. (1991). Biochemical topology: from vectorial metabolism to morphogenesis. Biosci. Rep. 11, 347–382 discussion: 382–385. 10.1007/BF01130213 1823595

[B46] Hooshangi S. Thiberge S. Weiss R. (2005). Ultrasensitivity and noise propagation in a synthetic transcriptional cascade. Proc. Natl. Acad. Sci. U.S.A. 102, 3581–3586 10.1073/pnas.0408507102 15738412 PMC552778

[B47] Hornung G. Barkai N. (2008). Noise propagation and signaling sensitivity in biological networks: a role for positive feedback. PLoS Comput. Biol. 4:e8 10.1371/journal.pcbi.0040008 18179281 PMC2174979

[B48] Huang S. Sultan C. Ingber D. (2006). “Tensegrity, dynamic networks, and complex systems biology: emergence in structural and information networks within living cells,” in Complex Systems Science in Biomedicine, eds Deisboeck T. S. Kresh J. Y. (Springer), 283–310 10.1007/978-0-387-33532-2_11

[B49] Jeong H. Mason S. P. Barabási A.-L. Oltvai Z. N. (2001). Lethality and centrality in protein networks. Nature 411, 41–42 10.1038/35075138 11333967

[B50] Jeong H. Tombor B. Albert R. Oltvai Z. N. Barabási A.-L. (2000). The large-scale organization of metabolic networks. Nature 407, 651–654 10.1038/35036627 11034217

[B51] Kardas D. Nackenhorst U. Balzani D. (2013). Computational model for the cell-mechanical response of the osteocyte cytoskeleton based on self-stabilizing tensegrity structures. Biomech. Model. Mechanobiol. 12, 167–183 10.1007/s10237-012-0390-y 22527364

[B52] Karr J. R. Sanghvi J. C. Macklin D. N. Gutschow M. V. Jacobs J. M. Bolival B. (2012). A whole-cell computational model predicts phenotype from genotype. Cell 150, 389–401 10.1016/j.cell.2012.05.044 22817898 PMC3413483

[B53] Kestler H. A. Kühl M. (2008). From individual Wnt pathways towards a Wnt signalling network. Philos. Trans. R. Soc. B Biol. Sci. 363, 1333–1347 10.1098/rstb.2007.2251 18192173 PMC2610122

[B54] Khalil A. S. Collins J. J. (2010). Synthetic biology: applications come of age. Nat. Rev. Genet. 11, 367–379 10.1038/nrg2775 20395970 PMC2896386

[B55] Kitano H. (2001). Foundations of Systems Biology. Cambridge, MA: MIT press

[B56] Kitano H. (2002a). Systems biology: a brief overview. Science 295, 1662–1664 10.1126/science.1069492 11872829

[B57] Kitano H. (2002b). Computational systems biology. Nature 420, 206–210 10.1038/nature01254 12432404

[B58] Klann M. Koeppl H. (2012). Spatial simulations in systems biology: from molecules to cells. Int. J. Mol. Sci. 13, 7798–7827 10.3390/ijms13067798 22837728 PMC3397560

[B59] Kobayashi H. Kærn M. Araki M. Chung K. Gardner T. S. Cantor C. R. (2004). Programmable cells: interfacing natural and engineered gene networks. Proc. Natl. Acad. Sci. U.S.A. 101, 8414–8419 10.1073/pnas.0402940101 15159530 PMC420408

[B60] Kramer B. P. Viretta A. U. Daoud-El Baba M. Aubel D. Weber W. Fussenegger M. (2004). An engineered epigenetic transgene switch in mammalian cells. Nat. Biotechnol. 22, 867–870 10.1038/nbt980 15184906

[B61] Kwok R. (2010). Five hard truths for synthetic biology. Nature 463, 288 10.1038/463288a 20090726

[B62] Lee H. DeLoache W. C. Dueber J. E. (2012). Spatial organization of enzymes for metabolic engineering. Metab. Eng. 14, 242–251 10.1016/j.ymben.2011.09.003 21946160

[B63] Liou S.-H. Chen C.-C. (2012). Cellular ability to sense spatial gradients in the presence of multiple competitive ligands. Phys. Rev. E Stat. Nonlin. Soft Matter Phys. 85:11904 10.1103/PhysRevE.85.011904 22400588

[B64] Long B. L. Rekhi R. Abrego A. Jung J. Qutub A. A. (2013). Cells as state machines: cell behavior patterns arise during capillary formation as a function of BDNF and VEGF. J. Theor. Biol. 326, 43–57 10.1016/j.jtbi.2012.11.030 23266714

[B65] Lu T. K. Khalil A. S. Collins J. J. (2009). Next-generation synthetic gene networks. Nat. Biotechnol. 27, 1139–1150 10.1038/nbt.1591 20010597 PMC2796205

[B66] Luengo-Oroz M. A. Duloquin L. Castro C. Savy T. Faure E. Lombardot B. (2008). “Can voronoi diagram model cell geometries in early sea-urchin embryogenesis?,” in Biomedical Imaging: From Nano to Macro, 2008. ISBI 2008. 5th IEEE International Symposium on, (Paris), 504–507

[B67] Ma'ayan A. Blitzer R. D. Iyengar R. (2005). Toward predictive models of mammalian cells. Annu. Rev. Biophys. Biomol. Struct. 34, 319–349 10.1146/annurev.biophys.34.040204.144415 15869393 PMC3035045

[B68] Marcotte E. M. (2001). The path not taken. Nat. Biotechnol. 19, 626–628 10.1038/90222 11433271

[B69] Michelotti N. Johnson-Buck A. Manzo A. J. Walter N. G. (2012). Beyond DNA origami: a look on the bright future of nucleic acid nanotechnology. Wiley Interdiscip. Rev. Nanomed. Nanobiotechnol. 4, 139 10.1002/wnan.170 22131292 PMC3360889

[B70] Miller M. Hafner M. Sontag E. Davidsohn N. Subramanian S. Purnick P. E. M. (2012). Modular design of artificial tissue homeostasis: robust control through synthetic cellular heterogeneity. PLoS Comput. Biol. 8:e1002579 10.1371/journal.pcbi.1002579 22829755 PMC3400602

[B71] Mitchell M. (1998). An Introduction to Genetic Algorithms. Cambridge, MA: MIT Press

[B72] Mobashir M. Schraven B. Beyer T. (2012). Simulated evolution of signal transduction networks. PLoS ONE 7:e50905 10.1371/journal.pone.0050905 23272078 PMC3521023

[B73] Mukherji S. van Oudenaarden A. (2009). Synthetic biology: understanding biological design from synthetic circuits. Nat. Rev. Genet. 10, 859–871 10.1038/nrg2697 19898500 PMC3138802

[B74] Neumann H. Neumann-Staubitz P. (2010). Synthetic biology approaches in drug discovery and pharmaceutical biotechnology. Appl. Microbiol. Biotechnol. 87, 75–86 10.1007/s00253-010-2578-3 20396881 PMC2872025

[B75] Nevozhay D. Zal T. Balázsi G. (2013). Transferring a synthetic gene circuit from yeast to mammalian cells. Nat. Commun. 4, 1451 10.1038/ncomms2471 23385595 PMC3573884

[B76] Ozbudak E. M. Thattai M. Kurtser I. Grossman A. D. van Oudenaarden A. (2002). Regulation of noise in the expression of a single gene. Nat. Genet. 31, 69–73 10.1038/ng869 11967532

[B77] Pedraza J. M. van Oudenaarden A. (2005). Noise propagation in gene networks. Science 307, 1965 10.1126/science.1109090 15790857

[B78] Pe'er D. (2005). Bayesian network analysis of signaling networks: a primer. Sci. STKE 2005:pl4 10.1126/stke.2812005pl4 15855409

[B79] Pfaendtner J. De La Cruz E. M. Voth G. A. (2010). Actin filament remodeling by actin depolymerization factor/cofilin. Proc. Natl. Acad. Sci. U.S.A. 107, 7299–7304 10.1073/pnas.0911675107 20368459 PMC2867716

[B80] Pothen J. J. Poynter M. E. Bates J. H. T. (2013). The inflammatory twitch as a general strategy for controlling the host response. J. Immunol. 190, 3510–3516 10.4049/jimmunol.1202595 23427255 PMC3608740

[B81] Purnick P. E. M. Weiss R. (2009). The second wave of synthetic biology: from modules to systems. Nat. Rev. Mol. Cell Biol. 10, 410–422 10.1038/nrm2698 19461664

[B82] Qutub A. A. Mac Gabhann F. Karagiannis E. D. Vempati P. Popel A. S. (2009). Multiscale models of angiogenesis: integration of molecular mechanisms with cell- and organ-level models. IEEE Eng. Med. Biol. Mag. 28, 14–31 10.1109/MEMB.2009.931791 19349248 PMC3077679

[B83] Rapaport D. C. (ed.). (2004). The Art of Molecular Dynamics Simulation. ISBN:0521825687. Cambridge, UK: Cambridge University Press, 564. 10.1017/CBO9780511816581

[B84] Ro D.-K. Paradise E. M. Ouellet M. Fisher K. J. Newman K. L. Ndungu J. M. (2006). Production of the antimalarial drug precursor artemisinic acid in engineered yeast. Nature 440, 940–943 10.1038/nature04640 16612385

[B85] Roze L. V. Chanda A. Linz J. E. (2011). Compartmentalization and molecular traffic in secondary metabolism: a new understanding of established cellular processes. Fungal Genet. Biol. 48, 35–48 10.1016/j.fgb.2010.05.006 20519149 PMC2949687

[B86] Ruder W. C. Lu T. Collins J. J. (2011). Synthetic biology moving into the clinic. Science 333, 1248–1252 10.1126/science.1206843 21885773

[B87] Ryan D. R. Hu J. Long B. L. Qutub A. A. (2013). “Predicting Endothelial Cell Phenotypes in Angiogenesis,” in ASME 2nd Global Congress on NanoEngineering for Medicine and Biology, (Boston, MA).

[B88] Scarselli F. Gori M. Tsoi A. C. Hagenbuchner M. Monfardini G. (2009). Computational capabilities of graph neural networks. IEEE Trans. Neural Netw. 20, 81–102 10.1109/TNN.2008.2005141 19129034

[B89] Schaller G. Meyer-Hermann M. (2005). Multicellular tumor spheroid in an off-lattice Voronoi-Delaunay cell model. Phys. Rev. E Stat. Nonlin. Soft Matter Phys. 71:51910 10.1103/PhysRevE.71.051910 16089574

[B90] Singhvi R. Stephanopoulos G. Wang D. I. C. (1994). Effects of substratum morphology on cell physiology. Biotechnol. Bioeng. 43, 764–771 10.1002/bit.260430811 18615800

[B91] Slusarczyk A. L. Lin A. Weiss R. (2012). Foundations for the design and implementation of synthetic genetic circuits. Nat. Rev. Genet. 13, 406–420 10.1038/nrg3227 22596318

[B92] Smolke C. D. Silver P. A. (2011). Informing biological design by integration of systems and synthetic biology. Cell 144, 855–859 10.1016/j.cell.2011.02.020 21414477 PMC3173940

[B93] Song H. Payne S. Gray M. You L. (2009). Spatiotemporal modulation of biodiversity in a synthetic chemical-mediated ecosystem. Nat. Chem. Biol. 5, 929–935 10.1038/nchembio.244 19915540 PMC2782429

[B94] Sozzani R. Benfey P. N. (2011). High-throughput phenotyping of multicellular organisms: finding the link between genotype and phenotype. Genome Biol. 12, 219 10.1186/gb-2011-12-3-219 21457493 PMC3129668

[B95] Spicher A. Michel O. Giavitto J.-L. (2011). “Interaction-based simulations for integrative spatial systems biology,” in Understanding the Dynamics of Biological System, eds Dubitzky W. Southgate J. Fuß H. (New York, NY: Springer), 195–231 10.1007/978-1-4419-7964-3_10

[B96] Stricker J. Cookson S. Bennett M. R. Mather W. H. Tsimring L. S. Hasty J. (2008). A fast, robust and tunable synthetic gene oscillator. Nature 456, 516–519 10.1038/nature07389 18971928 PMC6791529

[B97] Tabor J. J. Salis H. Simpson Z. B. Chevalier A. A. Levskaya A. Marcotte E. M. (2009). A synthetic genetic edge detection program. Cell 137, 1272 10.1016/j.cell.2009.04.048 19563759 PMC2775486

[B98] Takahashi K. Arjunan S. N. V. Tomita M. (2005). Space in systems biology of signaling pathways–towards intracellular molecular crowding *in silico*. FEBS Lett. 579, 1783–1788 10.1016/j.febslet.2005.01.072 15763552

[B99] Tanaka H. Yi T.-M. (2009). Synthetic morphology using alternative inputs. PLoS ONE 4:e6946 10.1371/journal.pone.0006946 19746161 PMC2735001

[B100] Thattai M. van Oudenaarden A. (2001). Intrinsic noise in gene regulatory networks. Proc. Natl. Acad. Sci. U.S.A. 98, 8614–8619 10.1073/pnas.151588598 11438714 PMC37484

[B101] Vendruscolo M. Dobson C. M. (2011). Protein dynamics: Moore's law in molecular biology. Curr. Biol. 21, R68–R70 10.1016/j.cub.2010.11.062 21256436

[B102] Walker D. C. Southgate J. (2013). The modulatory effect of cell–cell contact on the tumourigenic potential of pre-malignant epithelial cells: a computational exploration. J. R. Soc. Interface 10, 1–12 10.1098/rsif.2012.0703 23097504 PMC3565802

[B103] Wang B. Buck M. (2012). Customizing cell signaling using engineered genetic logic circuits. Trends Microbiol. 20, 376–384 10.1016/j.tim.2012.05.001 22682075

[B104] Wang B. Kitney R. I. Joly N. Buck M. (2011). Engineering modular and orthogonal genetic logic gates for robust digital-like synthetic biology. Nat. Commun. 2, 508 10.1038/ncomms1516 22009040 PMC3207208

[B105] Wang Z. Birch C. M. Sagotsky J. Deisboeck T. S. (2009). Cross-scale, cross-pathway evaluation using an agent-based non-small cell lung cancer model. Bioinformatics 25, 2389–2396 10.1093/bioinformatics/btp416 19578172 PMC2735669

[B106] Win M. N. Smolke C. D. (2008). Higher-order cellular information processing with synthetic RNA devices. Science 322, 456–460 10.1126/science.1160311 18927397 PMC2805114

[B107] Wodarz D. Hofacre A. Lau J. W. Sun Z. Fan H. Komarova N. L. (2012). Complex spatial dynamics of oncolytic viruses *in vitro*: mathematical and experimental approaches. PLoS Comput. Biol. 8:e1002547 10.1371/journal.pcbi.1002547 22719239 PMC3375216

[B108] Xie Z. Wroblewska L. Prochazka L. Weiss R. Benenson Y. (2011). Multi-input RNAi-based logic circuit for identification of specific cancer cells. Science 333, 1307 10.1126/science.1205527 21885784

[B109] Yang J. Meng X. Hlavacek W. S. (2010). Rule-based modelling and simulation of biochemical systems with molecular finite automata. IET Syst. Biol. 4, 453–466 10.1049/iet-syb.2010.0015 21073243 PMC3070173

[B110] Yeh B. J. Rutigliano R. J. Deb A. Bar-Sagi D. Lim W. A. (2007). Rewiring cellular morphology pathways with synthetic guanine nucleotide exchange factors. Nature 447, 596–600 10.1038/nature05851 17515921

[B111] Yingling M. O'Neill T. Skalak T. C. Peirce-Cottler S. (2005). A cellular automata model of circulating cell adhesion and transmigration in the microvaculature. IEEE Syst. Inform. Eng. Des. Symp. 1, 356–361 10.1109/SIEDS.2005.193280

[B112] You L. Cox R. S. Weiss R. Arnold F. H. (2004). Programmed population control by cell–cell communication and regulated killing. Nature 428, 868–871 10.1038/nature02491 15064770

[B113] Young E. Alper H. (2010). Synthetic biology: tools to design, build, and optimize cellular processes. J. Biomed. Biotechnol. 2010, 1–12 10.1155/2010/130781 20150964 PMC2817555

[B114] Zomaya A. Y. Kazman R. (2010). “Simulated annealing techniques,” in Algorithms and Theory of Computation Handbook, eds Atallah M. J. Blanton M. (Boca Raton, FL: CRC Press), 33.

